# All About Mitochondrial Eve: An Interview with Rebecca Cann

**DOI:** 10.1371/journal.pgen.1000959

**Published:** 2010-05-27

**Authors:** Jane Gitschier

**Affiliations:** Department of Medicine and Pediatrics, University of California San Francisco, San Francisco, California, United States of America

In unearthing the genetic history of human populations, the recent pace of discovery has been so rapid that we can lose sight of the impact made by a single paper. In a 1987 *Nature* article, Rebecca Cann and her co-workers, Mark Stoneking and the late Allan Wilson, painstakingly analyzed mitochondrial DNA purified from placentas that had been collected from women of many different ancestral origins. By comparing the mitochondrial DNA variants to each other, the authors produced a phylogenetic tree that showed how human mitochondria are all related to each other and, by implication, how all living females, through whom mitochondria are transmitted, are descended from a single maternal ancestor. Not only that, they localized the root of the tree in Africa. The report left a wake, still rippling today, that stimulated not just geneticists and paleo-anthropologists, but the layperson as well, especially as the ancestor was quickly dubbed “Mitochondrial Eve.” Indeed, the cover of *Newsweek* one year later depicted an Eden, replete with apple tree and serpent, but with the iconic blonde couple of Dürer now supplanted by an Adam and Eve of African descent.

I have always marveled at this paper, particularly as I had gotten to know Becky Cann (Image 1) when she was in the throes of completing this study and writing up the 40-something^th^ draft for its publication. At the time, I didn't appreciate the magnitude of what Becky had accomplished or the implications of the work. When the American Society of Human Genetics meeting was held in Honolulu in October, I arranged to interview her in her lab at the University of Hawaii to elicit her reflections on this discovery.

**Figure:**
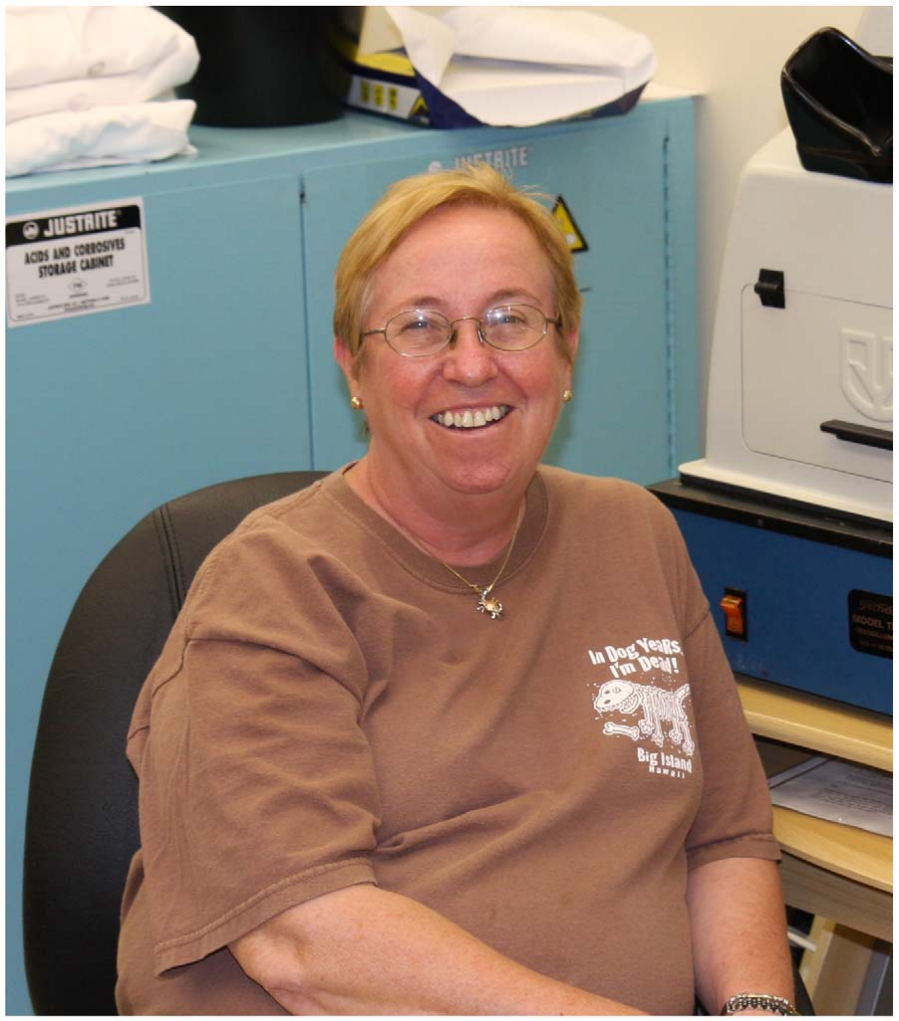
Rebecca Cann.

We met in the late afternoon, as students came by to submit their term papers under the 4 p.m. deadline. Becky has a kind of earth-mother quality about her, plain-spoken, clear-thinking, and supportive of her students. Still active in the field of human origins, Becky has also branched into avian work, examining the phylogeny of endangered species of native Hawaiian birds in Hakalau Forest National Wildlife Refuge with her husband and collaborator, Lenny Freed. The University's concrete campus lies deep within a canyon, its hard edges softened by mist and vegetation. Becky seems to fit right into the environment, fully grounded and far enough from the fray to follow her own path.

**Gitschier:** I noticed on your CV that you went to high school in San Francisco. Did you grow up there?

**Cann:** No, my parents moved from Des Moines, Iowa to San Francisco the summer before I started high school. My parents thought that they would gently move us into California by putting us in an all-girl Catholic High School, and they sent us to one on the edge of the Haight-Ashbury, not realizing the neighborhood it was in at the time and what we would be seeing on the way walking to school. It was quite a trip!

**Gitschier:** I'll bet—this was in the late 60s, right?

**Cann:** Yes—1967. I had a number of people ask me if I would like to try their pharmaceuticals on the way to school. They thought it was really funny to try to convince a little Catholic high school girl in a blazer, blue and white saddle shoes, a plaid skirt, and a white blouse to sample the wares.

**Gitschier:** But you made it through the school relatively unscathed–

**Cann:** Well, I think there are very few who make it through Catholic school, the last four years with only girls in their teenage years, unscathed.

**Gitschier:** OK, so it wasn't the Haight experience that was the problem.

**Cann:** I have a nun that spits fire on my windowsill.

**Gitschier:** Still! I had assumed from your last name that you might be Jewish.

**Cann:** My ex-husband was named Cann. I married for the first time in 1972, right after I graduated from Berkeley with my Bachelor's degree. I put an ex-husband through graduate school and then when he finished, I started graduate school.

**Gitschier:** Yes, I saw that you worked at Cutter Labs for five years after college. What was the evolution of that?

**Cann:** I got a Bachelor's degree in Genetics, and I thought I was interested in the genetics of behavior. I wanted to work on primates or humans, thinking that it would be a lot more interesting than fruit flies. But by the time I graduated, I came to see that the tools for doing human genetics were pretty crude. This was 1972.

**Gitschier:** Well, there was…

**Cann:** Nothing! Even restriction enzymes weren't there yet! So, I decided that since I really didn't have a good idea of what I wanted to do, I would find a job working as a quality control chemist—because I had the course work and I could get hired. Initially I began working nights so that I could take classes at Berkeley and read during the day. I used that time to branch out and think about how to put that interest in humans and human populations into a genetic context and to work explicitly at a molecular level.

I worked at night, right next to the mailroom, and I'd see all these journals coming in. Company scientists were talking about individuals varying in their ability to metabolize given compounds and how this influenced dose-response curves and that drugs that were developed for one population might not be effective for another. This woke me up to the fact that there really was this thing of personalized genomes that have a phenotypic effect. But how to get at the genotype?

I began to realize that there really was a significance for understanding those kinds of questions. But I was just learning how to be a good lab biologist at that time—how to be accurate, keep records, learn new technology, reproduce findings, and not to be frightened by the repetition of science.

**Gitschier:** That stood you well, then.

**Cann:** Oh, god—147 placental DNA preps later—yes!

**Gitschier:** OK, something makes you decide to go back to graduate school at Berkeley….

**Cann:** Yeah—restriction enzymes! I'm reading about them and taking classes, I learn about this crazy guy—Allan Wilson. Allan has this crazy idea that you can measure evolution by measuring mutation, but, in order to do that, you have to be able to find the same piece of DNA reproducibly in different individuals and in different species, so that you can do that kind of “molecular clock” comparison.

And he had already collaborated with an anthropology professor Vince Sarich. They had tried to do this indirectly with immunology, to look at the degree to which you could force an immune response. The idea being that the more closely related two individuals are to each other, the more parts of the immune system they would share. And with this technology they produced these trees that caused the paleontologists to just go nuts!

Then, suddenly there started to be this inkling that if you had a restriction enzyme you could do a Southern blot, and if you could do a Southern blot you could now start doing more individuals. You would be looking only at those genes for which you had a good probe, but you could do it a lot faster than you could produce individual amino acid sequences and there would be more variation there.

So, at that point, I decided this was going to be the wave of the future. There was a change in biotechnology, and I was goin' to grad school!

**Gitschier:** And you knew you wanted to work with Allan.

**Cann:** Allan or Vince. I didn't realize the degree to which they shared the laboratory at that point. And Vince was a real bench scientist—Allan wasn't. He had left the bench long ago. We used to joke that he didn't know which end of the Pipetman to put in the liquid. Allan was a professor of biochemistry and Vince was a professor of anthropology.

**Gitschier:** So, were you a graduate student in biochemistry?

**Cann:** This became an issue. I had been admitted to anthropology, and there was this question of whether I could function at the same level as biochemistry students. So there was this elitism right away. But Allan, once he watched me at the bench and knew something about my history, dismissed it. [I was] somebody who had worked at the bench as an industrial chemist and whose livelihood depended on doing the right thing. Very soon I was in the position of teaching his younger graduate students how to do bench science, and it was a big lab. At one point, Allan had 18 graduate students and six to eight post-docs. There was a lot of competition both for his time and also bench space. You had to be productive, ‘cause otherwise Allan wouldn't talk to you.

**Gitschier:** What year is this?

**Cann:** 1977. I was working on macaque serum proteins doing PAGE electrophoresis and stacked gel electrophoresis, looking at macaque species systematics—the shape of their phylogenetic tree. These are old-world primates that have spread out throughout the tropics and as they became isolated, they speciated.

**Gitschier:** So you're running serum proteins on a gel…

**Cann:** And you're staining with Coomassie blue, and you're asking what bands are shared between species. But you don't know which proteins are which.

So then we thought, let's use some hemoglobin Southern blots and let's see if we can sort this out. The idea of doing something very specific [with DNA blots] was very appealing: looking at the degree to which restriction maps matched or didn't match.

And then Wes Brown blew in. He was a post-doc from UCSF, with a Ph.D. from Cal Tech in Jerry Vinograd's lab. They did the original mitochondrial DNA isolations and had been isolating small viruses to chemical purity using CsCl density gradient centrifugation. Because Vinograd had just died, almost before he finished his thesis, Wes moved up here and was coming over to write grants with Allan. He knew that Allan was interested in timing and clocks and evolution. He said, “By the way, you know you could take this purified fraction of mitochondrial DNA away from the genomic DNA and then bust it open with restriction enzymes and study that—and this stuff changes really fast!”

So Wes and Allan were writing these grants, and Wes had a paper on 21 isolated mitochondrial fractions that he had gotten from placentas from labor and delivery. And they showed this incredible variation! But the medical records were disassociated; he didn't know much about the donors [i.e., their ethnicities].

It got to the point where you could end-label [DNA fragments with ^32^P]. So he was taking Klenow fragment, end-labeling his pure fractions after restriction enzyme digestion, and running them out on long gels to construct a physical map. You'd figure out the size of the fragments, knowing the total size of the [mitochondrial] genome, starting with the 6-base cutters, and map the mitochondrial genome.

Wes's big contribution was totally changing over Allan's lab from a protein-biochemistry approach to studying evolution to a DNA-based approach. He brought not only the restriction technology, but also cloning mitochondrial fragments. We did Sanger sequencing and showed exactly the spectrum of mutations, and that even though there were essential genes in the mitochondria, they still changed faster than the same classes of genes in the nuclear genome.

**Gitschier:** What prompted *you* to make the switch to mitochondria?

**Cann:** I saw that that was a potential tool to break open the question of human variation.

All this time I was taking graduate seminars. I took all the human anatomy, taught by the physical anthropologists and Tim White, who was part of the Laetoli footprint team in the late 70s. He was the anatomical expert during the Lucy discovery, too.

So I'd come from the lab, and I hear all this “yammer yammer yammer” about 2- and 3-million-year-old fossils and which lineage goes where. And from my understanding of the human fossil record, we hit Cro-magnon and nobody knows how that's related to Neanderthal. And *Homo erectus*. What happened there?

So talking to Allan and to Wes, and reading and arguing a lot, [we thought] potentially you could expand this view of human evolution in that time period by doing a mitochondrial analysis, because there was enough variation there. If you used your average nuclear gene, there wasn't enough resolution. The thinking was that this technology would give us enough differences between populations to start asking whether there is a most-recent common ancestor that is different for this group vs. that group, and how these older archaic populations in Asia and Africa are related to the modern people.

**Gitschier:** But did you imagine that you could actually get mitochondrial DNA from the archaic people?

**Cann:** Why not? Some of the fossils I was looking at had organic material in them. And people had been trying to get stuff out of blood on tools. So, who knew what you could get out of a fossil? At the same time, Allan came back from sabbatical and he had a little piece of a frozen Siberian mammoth that they had on exhibit; he had shaved off the heel. And he had people going to the gem shows to pick up amber.

Once the mitochondrial thing took off, the reason people started thinking about ancient material was that we knew mitochondrial DNA was such a large fraction of DNA in the cell, so we knew if anything was going to survive, it wasn't going to be single copy nuclear genes—it would be something in the mitochondria.

It was a fertile time. Lots of ideas floating. Allan used to say, “Keep having ideas—some will be good, some will be crap. Just keep thinking them up.”

**Gitschier:** OK. I want to talk to you about a paper by Cann and Wilson in *Genetics* in 1983. You've made DNA from 110 human cell lines or placentas, including from people of African descent, and you have this tree. But this tree doesn't look like the next tree in the big paper [*Nature* 1987]. You don't show African origins in the 1983 paper; in fact, you say there is no strong correlation with race or geography, consistent with multiple origins for length mutations. This ultimately is not correct, is it?

**Cann:** Unh unh….

**Gitschier:** So, what I'm trying to figure out is what happened between these two papers.

**Cann:** PAUP, the new phylogeny program—“phylogenetic analysis using parsimony”—devised by David Swofford. In the earlier papers, we used Fitch-Margoliash trees, which are *distance* trees.

There are two ways that you can draw a pattern of relatedness between two individuals. One way is to look at similarity, and just take that measure—just add up all the differences.

**Gitschier:** So let's see if I get this. In the 1983 paper, you have a large matrix of differences between the 110 people…

**Cann:** [Nods] Or, instead, if you have DNA sequence information or a good enough restriction map, you can see what base had to have changed. The other thing that had happened was the Cambridge reference sequence [for mitochondrial DNA] had been published, so I could take my restriction map and match where the restriction enzyme site was on the reference DNA [sequence]. I could figure out—in order to generate this site or lose this site—what that change had to be. Suddenly, you went from being able to extract not just how different or similar two individuals were and put them on a distance tree, but also, with the sequence, you can use a parsimony principle and say what changes are present in two or more individuals.

You are trying to use the information with the assumption that the mutation happened once and only once. And then successively build up these blocks of sequence that have to be more closely related to each other, with that assumption, by parsimony.

Originally the PAUP algorithm couldn't take so many samples—originally it was 30 by 30, and then 50 by 50. And by the 1987 paper, PAUP could take 150.

**Gitschier:** And you have 147 samples.

**Cann:** Yes, in my thesis, in 1982, I had used PAUP along with Fitch-Margoliash and Neighbor Joining—the distance type of algorithms—and tried to compare them. I had to use random sub-draws of 30 individuals with the parsimony tree—because that was the biggest matrix PAUP would take—and I would try to see whether randomly generating those matrices with parsimony gave me something different than the distance trees.

And they did, but I couldn't prove it, just randomly pulling and having variation so that the ethnicities were stratified across 28 Asians and 2 Africans, for example. I was still getting a pattern that was different from what the distance trees gave. But I couldn't convince Allan that it was really showing the African origin. I couldn't convince him.

[Luca] Cavalli-Sforza had published a paper with Doug Wallace saying that Asia is the origin. Other people, like [Milford] Wolpoff in Michigan, were arguing that the mitochondrial trees couldn't possibly ever be right, and I, in particular, could never be right ‘cause the greatest human geneticist living had just published this other tree that showed human ancestry in Asia.

**Gitschier:** Where did they go wrong with that?

**Cann:** They were using distance trees. They had a really long branch to the Africans in their sample, but Cavalli believed that human origins were Asian, and that Africans just had this wild mutation rate because their environment was so bizarre. [He thought] if you are really going to root the human tree, Asia was a better place to do it.

There really wasn't good evidence, other than thinking that modern humans couldn't have evolved in Africa. Biologically, they had evidence that should have placed it in Africa. And the Japanese geneticists, like Masatoshi Nei, saw that and called it. They said, “You can't defend this. There is no good archeological evidence that would force you to put that root there [in Asia].”

**Gitschier:** So these samples that you used were the same ones that you had looked at before.

**Cann:** There were some additions. I got additional Australian aborigines, finally. I had been waiting for about 40 more to come. It was now 1984, I was in my first post-doc and I was cloning and sequencing some of them. Mark Stoneking came to the lab and Allan suggested that Mark do this mapping on the additional Australians and the Papua New Guinea highlands, which was his thesis. So, Mark contributed those to this paper.

**Gitschier:** At what point is Allan on board?

**Cann:** We wrote the paper and submitted it in late '85, and it got held up in review for over a year in *Nature*, because the Brits didn't want it to be published.

**Gitschier:** Why not?

**Cann:** There was a certain group that wanted to publish their phylogeny of globins.

**Gitschier:** Ah. Why didn't you just pull it and sent it to *Science*?

**Cann:** I think Allan wanted the prestige of *Nature*. He's a New Zealander, proud of it, and wanted to show them up. He published a lot of papers there. We talked about moving it, but he had faith that eventually they would understand the value of it. I think he was reticent to talk about the personalities of the people involved.

Whenever I'd get a review back he'd say, “Don't worry about who reviewed it. It's not a positive thing to be thinking about, and it won't help you make a better paper.” He knew there were some really sharp personalities that were directed at a woman—an American—and an upstart Colonial. He didn't want me to start thinking that this is what I was going to face in science.

He was a real Marxist. He knew how hard it was for women to get going in science. He'd seen it—his lab was a haven for the women in the program and the male professors from other labs would joke that Allan had all the women. What was so special about Allan? He was gender blind. If you had a good idea, it was a good idea.

**Gitschier:** But back to the tree—you already suspected that the tree was going to look this way.

**Cann:** I didn't know for sure that this was how it was going to look. Mathematically, given all these samples, there were lots of possibilities. A universe of trees! But this tree could be reproduced—the order of entry could influence the outcome, so we would reorder the entry, and run against subsets of individuals. This was a plausible tree.

Eventually Allan was comfortable with the idea and that it could be defended. We didn't go out on the limb and say it was the *best* tree, but it was a tree with a high likelihood of being correct and it was consistent with a lot of other data —anatomically modern fossil forms in South Africa around 200,000 years ago. Then White found another fossil in East Africa from around the same time. And the Middle East fossils were re-dated at 110,000 years.

**Gitschier:** When it came out, this 1987 paper must have changed your life.

**Cann:** Not for the good, sometimes. I got a lot of hate mail, crank mail, some with strange scrawling notes. I even got a visit from the FBI after the Unabomber attacks. I got random calls in the middle of the night, and people on flight layovers wanted to talk. I was unprepared for this role as the molecular person questioning the fossils – and for people like Wolpoff saying these archaic people evolved into modern people, or that I had studied African Americans, not real Africans…

It made me mad because people were doing the same thing with birds and lizards and fish and they weren't taking anywhere near the amount of crap I was taking. I could see it was only because I was talking about humans. These arguments raised so much emotion, and that really depressed me.

**Gitschier:** What was Allan's reaction to the press on this?

**Cann:** He was bemused. People had two reactions: either (1) they knew it all along, or (2) it can't possibly be right. So he was trying to find a predictor of who was going to say what to him. Would it correlate with any other prejudice he had based on past interactions or personality type?

I remember a discussion over dinner one night about three years after it was published. There were a number of population geneticists, like Alan Templeton, who still haven't resolved in their minds that this African origin idea could be correct. He continues to write stuff about this. And it's not that I don't want to listen to criticism, because there were things that obviously we didn't have the answers to when this was written. Mitochondrial sequencing has shown certain areas that will generate distortions, and we didn't have all the samples we would like to have had. There were some leaps of faith.

Sometimes I've heard Luca talk and say, “Well they got the right answer, but they didn't know why they got it.” And I always thought that was dismissive. I had a pretty good idea why I got the right answer!

